# Liver diseases as a novel risk factor for delirium in the ICU–Delirium and hepatic encephalopathy are two distinct entities

**DOI:** 10.1371/journal.pone.0276914

**Published:** 2022-11-22

**Authors:** Alexander Denk, Karolina Müller, Sophie Schlosser, Klaus Heissner, Karsten Gülow, Martina Müller, Stephan Schmid

**Affiliations:** 1 Department of Internal Medicine I, Gastroenterology, Hepatology, Endocrinology, Rheumatology and Infectious diseases, University Hospital Regensburg, Regensburg, Germany; 2 Center for Clinical Studies, University Hospital Regensburg, Regensburg, Germany; Medizinische Fakultat der RWTH Aachen, GERMANY

## Abstract

**Background:**

Delirium prevalence is high in critical care settings. We examined the incidence, risk factors, and outcome of delirium in a medical intensive care unit (MICU) with a particular focus on liver diseases. We analyzed this patient population in terms of delirium risk prediction and differentiation between delirium and hepatic encephalopathy.

**Methods:**

We conducted an observational study and included 164 consecutive patients admitted to an MICU of a university hospital. Patients were assessed for delirium using the Confusion Assessment Method for ICUs and the Richmond Agitation-Sedation Scale (RASS). On admission and at the onset of delirium Sequential Organ Failure Assessment (SOFA) score was determined. A population of patients with liver disease was compared to a population with gastrointestinal diseases. In the population with liver diseases, hepatic encephalopathy was graded according to the West Haven classification. We analyzed the incidence, subtype, predisposing, precipitating, and health-care setting-related factors, treatment, outcome of delirium and the association between delirium and hepatic encephalopathy in patients with liver diseases.

**Results:**

The incidence of delirium was 32.5% (n = 53). Univariable binary regression analyses adjusted by the Holm-Bonferroni method showed that the development of delirium was significantly determined by 10 risk factors: Alcohol abuse (p = 0.016), severity of disease (Simplified Acute Physiology Score (SAPS) II, p = 0.016), liver diseases (p = 0.030) and sepsis (p = 0.016) compared to the control group (gastrointestinal (GI) diseases and others), increased sodium (p = 0.016), creatinine (p = 0.030), urea (p = 0.032) or bilirubin (p = 0.042), decreased hemoglobin (p = 0.016), and mechanical ventilation (p = 0.016). Of note, we identified liver diseases as a novel and relevant risk factor for delirium. Hepatic encephalopathy was not a risk factor for delirium. Delirium and hepatic encephalopathy are both life-threatening but clearly distinct conditions. The median SOFA score for patients with delirium at delirium onset was significantly higher than the SOFA score of all patients at admission (p = 0.008). Patients with delirium had five times longer ICU stays (p = 0.004) and three times higher in-hospital mortality (p = 0.036). Patients with delirium were five times more likely to be transferred to an intensive medical rehabilitation unit for post-intensive care (p = 0.020). Treatment costs per case were more than five times higher in patients with delirium than in patients without delirium (p = 0.004).

**Conclusions:**

The 10 risk factors identified in this study should be assessed upon admission to ICU for effective detection, prevention, and treatment of delirium. Liver diseases are a novel risk factor for delirium with a level of significance comparable to sepsis as an established risk factor. Of note, in patients with liver diseases delirium and hepatic encephalopathy should be recognized as distinct entities to initiate appropriate treatment. Therefore, we propose a new algorithm for efficient diagnosis, characterization, and treatment of altered mental status in the ICU. This algorithm integrates the 10 risk factor prediction-model for delirium and prompts grading of the severity of hepatic encephalopathy using the West Haven classification if liver disease is present or newly diagnosed.

## Introduction

With an incidence of 20–80%, delirium is the most common psychiatric disorder in intensive care units (ICU) [1–5]. According to the Diagnostic and Statistical Manual of Mental Disorders (DSM-5), delirium is defined as a psychiatric disorder due to an organic cause [6]. Delirium, which is characterized by acute onset and fluctuations throughout the day, is associated with both attention and concentration disorders as well as with other cognitive disorders [7, 8]. According to the Richmond Agitation Sedation Scale (RASS) score, delirium can be classified into three subtypes: hypoactive, hyperactive, and mixed type delirium [9–12].

Despite the increasing number of studies on delirium published in recent years [2], the pathophysiology of delirium is still not fully understood. The prevailing assumption is that delirium has a multifactorial etiology with different pathophysiologies, which mutually determine and influence each other [13–15]. These pathophysiologies include neurotransmission disorders [16, 17], elevated cortisol levels as a result of chronic stress [18, 19], systemic inflammation [20–22], hypoxia [22, 23], an aging central nervous system [24–28], and disorders of melatonin metabolism affecting the sleep-wake cycle [13, 29].

The risk of delirium is determined by (i) predisposing risk factors, (ii) precipitating risk factors and (iii) health-care setting-related risk factors [1]. Predisposing risk factors comprehend the characteristics of patients, for example increased age, dementia, frailty, comorbidities such as cardiovascular and renal disease, alcohol abuse, reduced nutritional status and visual or hearing impairment. Precipitating risk factors cover a range of insults, taking in the acute medical illness, surgery, dehydration, sepsis, hypoglycemia, pain and medication changes among others. In addition to these predisposing and precipitating factors, specific health-care setting-related factors, for example mechanical ventilation or therapeutic interventions, are risk factors for delirium [1, 13, 30–37]. In the (M)ICU setting typically more than one risk factor is present and total risk depends on the number of risk factors in each patient. A meta-analysis examining delirium-associated mortality in different subpopulations (ICU, medical, post-acute, and surgical patients) showed that data on delirium depend on the setting of their collection [38].

Delirium remains under-diagnosed. According to the current literature, less than half of delirium cases in hospital are detected [1, 39]. Especially in approaching the challenges in achieving better rates of delirium detection, it is important to study different settings [40, 41]. Delirium is a common medical emergency and has been assessed after elective surgery, in patients with cardiac diseases and in elderly patients. Data on patients with liver diseases are missing.

Of note, a recent nationwide population-based study in Germany by Gu et al. showed that cirrhosis represents a considerable healthcare burden, as shown by the increasing in-hospital mortality. Of clinical relevance, the authors show that alcohol related cirrhosis and complications as well as non-alcoholic fatty liver diseases are significantly on the rise [42]. The authors conclude that better management strategies are warranted.

In our study the population of patients with liver diseases has been specifically investigated in terms of delirium risk prediction and the differentiation of delirium and hepatic encephalopathy as major determinants of mortality. There are major challenges in the implementation of effective detection, prevention and treatment of delirium and differential diagnosis of hepatic encephalopathy (HE) in patients with liver diseases [43].

Therefore, we have assessed delirium risk in patients on an MICU with focus on hepatology, gastroenterology and infectious diseases. To the best of our knowledge, no study to date has investigated the outcome and potential risk factors of delirium in a population of patients with liver diseases.

The burden of liver disease is significant nationally as shown by Gu et al. and worldwide [42, 44]. Progression of liver disease to fibrosis and cirrhosis and decompensation associated with critical illness is a major cause of mortality in this population. Acute-on-chronic liver failure is a recently described entity diagnosed in patients with chronic liver disease and a combination of hepatic and extrahepatic organ failures (kidney, respiratory, circulatory, brain) [45–48].

Brain failure is defined as grade 3 or 4 HE diagnosed according to the West Haven classification. Grade 3/4 HE has been shown to be independently associated with mortality, regardless of other organ failures [49]. The pathogenesis of HE is related to hyperammonemia, gut microbial dysbiosis and systemic inflammation in the setting of precipitating factors like infections or medication. It is important to consider other causes of altered mental status in addition to HE because patients with chronic liver disease are also affected by mental changes related to infections, medications, electrolyte imbalances, alcohol and illicit drugs, and strokes [46]. HE and delirium are life-threatening conditions and making the right diagnosis early is key to specific treatment schedules and a better outcome.

Therefore, our objectives were to study delirium in a medical ICU setting with focus on a population of patients with liver diseases regarding (i) incidence, (ii) motoric subtypes, (iii) predisposing risk factors, (iv) precipitating risk factors, (v) health-care setting-related risk factors (vi) differential diagnosis hepatic encephalopathy, (vii) clinical outcome, and (viii) economic consequences.

## Material and methods

### Study design and patient characteristics

To investigate incidence, subtype, predisposing risk factors, precipitating risk factors, health-care setting-related risk factors, economic consequences and outcome of delirium and HE in an MICU setting, the study included all patients treated at a MICU of a German University hospital between February and August 2017. A specific focus of the study was to establish a new algorithm for the diagnosis, characterization, and treatment of altered mental status in the ICU that allows to differentiate the life-threatening conditions sepsis, delirium, and hepatic encephalopathy. The study was approved by the Ethical Committee of the University of Regensburg, Regensburg, Germany. Written informed consent was obtained. In total, 250 patients were assessed for eligibility.

### Assessment for coma

Inclusion criteria were a Richmond Agitation-Sedation Scale (RASS) score of ≥ -3 and an ICU stay of at least 24 h. The RASS score was used to assess the level of patient alertness or agitation and the subtype of delirium [9]. In accordance with the DSM-5 criteria and the Statement of the Board of the European Delirium Association and the American Delirium Society, patients with all states of altered arousal except coma were included in our study (RASS score ≥ -3) [7, 50]. 30 patients were in coma and were excluded, 39 patients were excluded due to ICU stay < 24 h and 17 patients were excluded due to missing data. Accordingly, 164 patients were included in the study.

### Assessment for delirium

**Fig 1:**
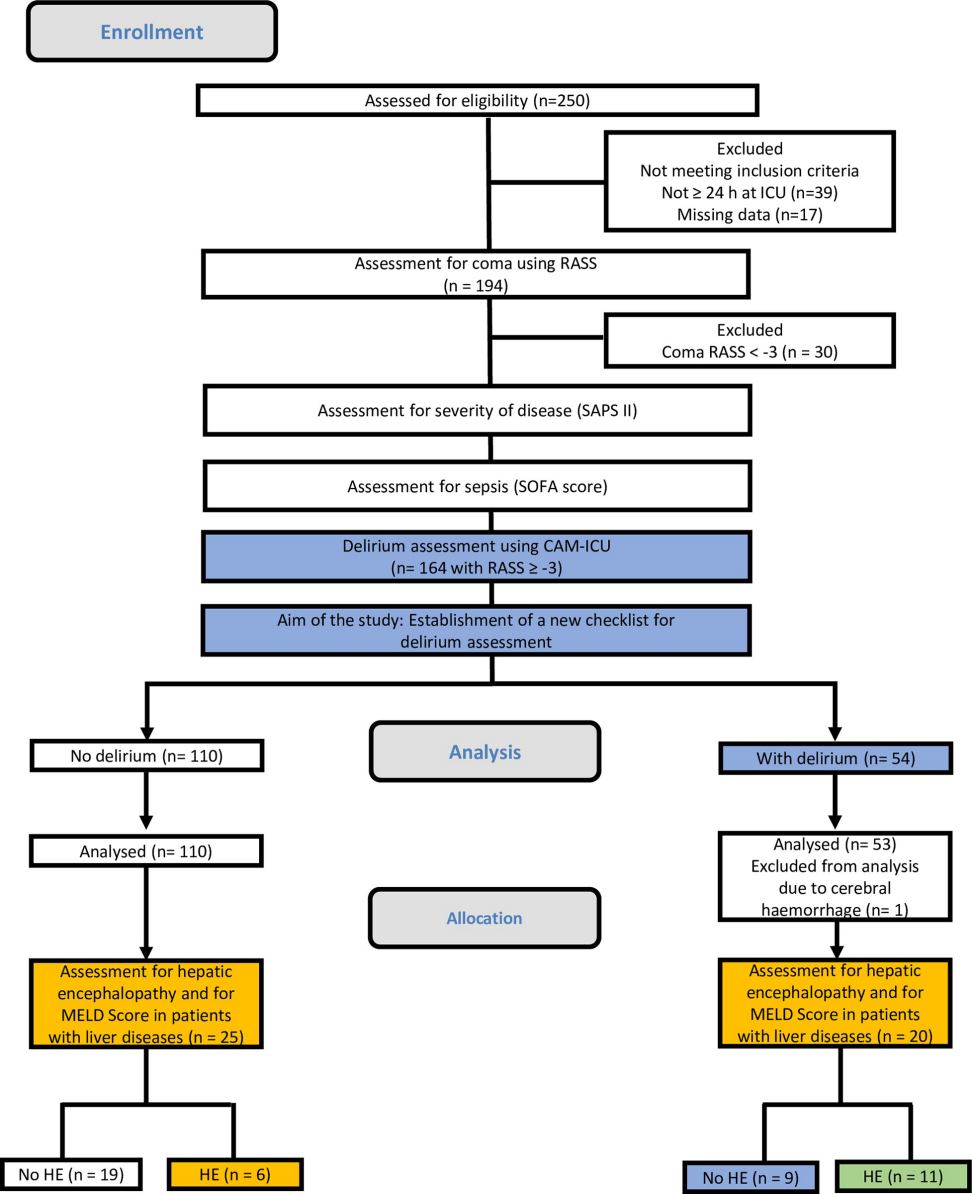
Study design and inclusion of patients. RASS = Richmond Agitation Sedation Scale, SAPS II = Simplified Acute Physiology Score II, SOFA score = Sepsis-related organ failure assessment score, CAM-ICU = Confusion Assessment Method for Intensive Care Unit, HE = Hepatic encephalopathy.

The delirium status was evaluated by means of the Confusion Assessment Method for ICUs (CAM-ICU) [51–54] out of 164 patients were diagnosed with delirium. Due to cerebral hemorrhage one patient was excluded [7]. Overall, 53 patients with delirium of 163 patients in total were included in the analyses (Fig 1).

Furthermore, a risk factor assessment was performed, which included the clinically most relevant predisposing risk factors, precipitating risk factors and health-care setting-related risk factors for delirium, consistent with the current literature [1].

Predisposing (= premorbid) risk factors for delirium are factors included in the background characteristics of patients. In our study these risk factors included age, sex, BMI, tobacco use, and alcohol abuse. Tobacco use was defined as daily consumption. Alcohol abuse was defined as > 12 g alcohol/day for women and > 24 g alcohol/day for men [54].

Precipitating factors for delirium comprise acute insults. Therefore, acute illnesses such as liver diseases or sepsis, severity of disease (Simplified Acute Physiology Score [SAPS] II), electrolyte imbalance, and other laboratory parameters were included.

In addition to predisposing and precipitating risk factors, specific health-care setting-related factors, such as mechanical ventilation and therapeutic interventions, which are known risk factors for hospital-acquired delirium were addressed (Tables 1 and 3).

**Table 1:** Patient characteristics and risk factors.

	n	Patients without delirium n = 110	Patients with delirium n = 53
**Predisposing risk factors**			
**Age (med, IQR)**	163	60.0 (42.5–69.0), n = 110	63.0 (54.0–73.0), n = 53
**Sex**	163		
Men (%)	93	59 (63.4)	34 (36.6)
Women (%)	70	51 (72.9)	19 (27.1)
**BMI (med, IQR)**	161	24.8 (21.6–28.0), n = 109	25.9 (23.0–29.1), n = 52
**Tobacco use**	153		
No (%)	91	25 (27.5)	66 (72.5)
Yes (%)	62	23 (37.1)	39 (62.9)
**Alcohol abuse**	157		
No (%)	99	79 (79.8)	20 (20.2)
Yes (%)	58	27 (46.6)	31 (53.4)
**Precipitating risk factors**			
**SAPS II score (med, IQR)**	153	13.0 (8.5–20.0), n = 102	23.0 (16.0–31.5), n = 51
**Acute medical illness**	163		
Liver diseases (%)	45	25 (55.6)	20 (44.4)
Sepsis (%)	27	12 (44.4)	15 (55.6)
GI diseases and others (= control group) (%)	91	73 (80.2)	18 (19.8)
Hepatic encephalopathy^1^	17	6 (35.3)	11 (64.7)
**Healthcare setting related risk factors**			
**Mechanical ventilation**	161		
No (%)	117	90 (76.9)	27 (23.1)
Yes (%)	44	18 (40.9)	26 (59.1)
Interventions^2^	140		
No (%)	64	47 (44.8)	17 (48.6)
Yes (%)	76	58 (55.2)	18 (51.4)

Presentation of patient characteristics, predisposing (age, sex, BMI, tobacco use, alcohol abuse), precipitating (acute medical illness, SAPS II score), and health-care setting-related (interventions and surgeries, mechanical ventilation) related risk factors.

^1^Hepatic encephalopathy defined according to the West Haven classification

^2^ n = 55 (72.4%) of the interventions were GI endoscopies.

BMI = Body Mass Index; med = Median; GI = gastrointestinal; IQR = Interquartile range; SAPS = Simplified Acute Physiology Score.

In patients without delirium, we analyzed the values of the SAPS II score and laboratory parameter recorded on admission to the ICU, and in patients with delirium, the values measured at the onset of delirium. In addition, we assessed whether the patients had an increased risk of developing delirium within 48 hours after termination of mechanical ventilation (Table 1).

The acute medical illness was categorized into three disease entities, (i) liver diseases, (ii) sepsis, and (iii) gastrointestinal (GI) diseases and others. This comparison was performed to evaluate whether patients with liver diseases or sepsis could have an increased risk for delirium compared to GI diseases or others (control group). Patients with liver diseases and sepsis, i.e., two conditions, were diagnosed acute-on-chronic liver failure (ACLF) and were therefore categorized in group i (liver diseases). In the sepsis group (ii) there were no patients with underlying liver diseases, and we have given the causes of sepsis in Table 2. Table 2 provides a detailed list of diseases in the respective subgroups. Furthermore, we aimed to establish an algorithm to distinguish delirium from HE in the subgroup of patients with liver diseases.

**Table 2:** List of diseases of patients in subgroups i, ii, and iii.

Liver diseases (i) n = 45	n(%)	Sepsis (ii) n = 27	n(%)	GI diseases and others (iii) n = 91	n(%)
Acute decompensated liver cirrhosis	30 (66.7)	Abdominal sepsis	14 (51.9)	Gastrointestinal bleeding	15 (16.5)
Acute on chronic liver failure	9 (20.0)	Pulmonary sepsis	10 (37.0)	Pancreatitis	12 (13.2)
Acute liver failure	5 (11.1)	Cutaneous sepsis	2 (7.4)	Motility disorders	6 (6.6)
Acute Budd-Chiari syndrome	1 (2.2)	Urogenital sepsis	1 (3.7)	Gastrointestinal cancer	4 (4.4)
				GI-Perforation	3 (3.3)
				Foreign body ingestion	3 (3.3)
				Intoxication	6 (6.6)
				Parasitic diseases	1 (1.1)
				Autoimmune and metabolic diseases	13 (14.3)
				Acute exacerbations of chronic obstructive pulmonary disease	12 (13.2)
				Cardiovascular disease	8 (8.8)
				Kidney diseases	4 (4.4)
				Neurologic/psychiatric diseases	4 (4.4)

List of diseases of patients in subgroups i (liver diseases), ii (sepsis), and iii (GI diseases and others). Sepsis was defined according to The Third International Consensus Definitions for Sepsis and Septic Shock (Sepsis-3) [62].

### Assessment for hepatic encephalopathy in the subgroup of patients with liver diseases

In the subgroup of patients with liver diseases HE has been diagnosed according to the West Haven classification, which is the gold standard for the assessment of HE [55, 56]. Neurological symptoms of HE as asterixis (= flapping hand tremor), which have a good inter-rater reliability, were additionally recorded [46, 57].

### Assessment of Model for End-Stage Liver Disease (MELD) score

The MELD score, a well-established indicator of the mortality of patients with end-stage liver disease, was calculated for each patient with liver diseases using the following equation [58, 59]:

MELD score = 9.57 x ln(serum creatinine) + 3.78 ln(total bilirubin) + 11.2 x ln(international normalized ratio) + 6.43

### Assessment of sequential organ failure assessment (SOFA) score

Admission SOFA score for all patients and for patients with delirium SOFA Score at onset of delirium was determined [60–64]. The respiratory SOFA component was determined according to [65, 66].

### Pharmacological treatment of delirium

Expert consensus supports a limited role for drugs in the treatment of patients with delirium [67]. Therefore, the medication that patients received on average per day during delirium was analyzed and compared to the medication of patients without delirium. A subgroup analysis was performed for patients with liver diseases. Chi-squared test was used to test for significance.

### Assessment of the outcome and the consequences for the health system

Patient outcome was assessed by means of length of the ICU stay, in-hospital mortality, and transfer to an intensive medical rehabilitation unit for post-intensive care. In addition, costs for the healthcare system were calculated using German diagnosis-related group (G-DRG)-based reimbursement.

### Statistical analyses

Data were self-reported by the patients, obtained from the in-house patient data management system Metavision (iMDsoft, Dusseldorf, Germany) and from the SAP hospital information system (SAP, Walldorf, Germany). Analyses were performed using absolute and percentual frequency (n, %) and median including interquartile range (IQR). Due to the number of patients with delirium, univariable analyses were conducted. Mann-Whitney-U-tests for independent samples were used to compare ICU outcomes between patients with and without delirium. The effect of 17 demographic and clinical parameters on the development of delirium was analyzed using univariable binary logistic regressions. For patients with liver disease, subgroup analyses were performed regarding the diagnosis of HE and the respective pharmacological treatment. Odds ratios (OR) including 95% confidence interval (CI) are presented. Statistical analyses were conducted with SPSS Statistics 26 (SPSS Inc, Chicago, Illinois). The level of significance was set at p_two-sided_ ≤ 0.050. Adjustments for multiple testing were performed by the Holm-Bonferroni method for risk factors of delirium in an MICU (Table 4) as well as for clinical outcome and health-care expenditure (Table 5).

## Results

### Incidence of delirium, hepatic encephalopathy, and patient characteristics

**Fig 2:**
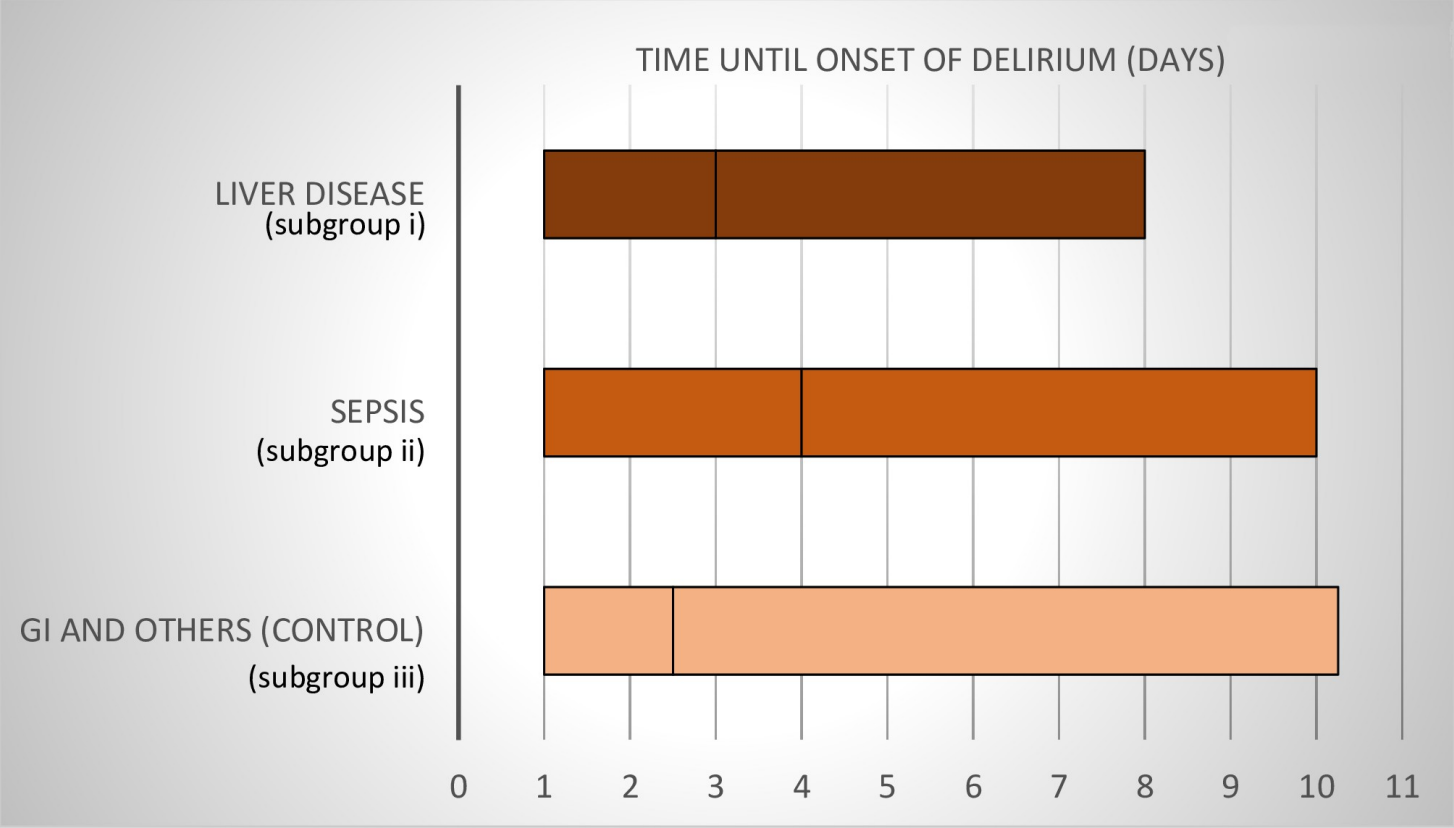
Median time until onset of delirium in the subgroups of patients with liver diseases, sepsis, and GI diseases and others (control). GI = gastrointestinal.

Of the 163 patients included, 53 (32.5%) developed delirium. Median age of all patients was 60.0 years (IQR = 50.0–70.0), median BMI 25.4 kg/m^2^ (IQR = 22.5–28.4), and median SAPS II score 17.0 (IQR = 10.0–23.0). The majority of the patients were men (57%). The percentages for substance abuse were 36.9% for alcohol and 40.5% for tobacco use. The median time until onset of delirium was 3.0 days (IQR = 1.0–9.5). There was no significant difference between the subgroups (liver diseases: 3.0 days (IQR = 1.0–8.0), sepsis: 4.0 days (IQR = 1.0–10.0) and GI diseases and others: 2.5 days (IQR 1.0–10.25), p = 0.398). The median time until onset of delirium in the different subgroups is presented in Fig 2.

Furthermore, Tables 1 and 2 illustrate the characteristics of the patients with and without delirium, including their demographic data, acute medical illness, and therapeutic interventions grouped by predisposing-, precipitating-, and health-care setting-related risk factors. Furthermore, the subgroup of patients with liver diseases, who presented with hepatic encephalopathy with or without concomitant delirium is depicted in Table 1. Table 3 shows clinically relevant laboratory parameters including clinical chemistry and blood count.

**Table 3:** Laboratory parameters (med, IQR).

	n	Patients without delirium n = 110	Patients with delirium n = 53
**Sodium** (mmol/l)	162	139.0 (136.0–142.0), n = 110	143.0 (139.0–142.0), n = 52
**Hemoglobin** (g/dl)	162	10.7 (8.5–13.0), n = 110	8.7 (7.8–9.9), n = 52
**Creatinine** (mg/dl)	154	0.89 (0.70–1.4), n = 108	1.3 (0.71–2.2), n = 46
**Urea** (mg/dl)	162	38.0 (25.8–85.0), n = 110	71.5 (39.0–112.0), n = 52
**Bilirubin** (mg/dl)	161	0.80 (0.45–1.8), n = 109	1.25 (0.70–6.9), n = 52
**CRP** (mg/l)	147	36.1 (14.6–109.8), n = 96	57.2 (35.7–92.9), n = 51

Selected, clinically relevant laboratory parameter from clinical chemistry and blood count included in the evaluation as precipitating risk factors.

CRP = C-reactive protein; med = Median; GI = gastrointestinal; IQR = Interquartile range; SAPS II = Simplified Acute Physiology Score.

### Motoric subtype of delirium

**Fig 3:**
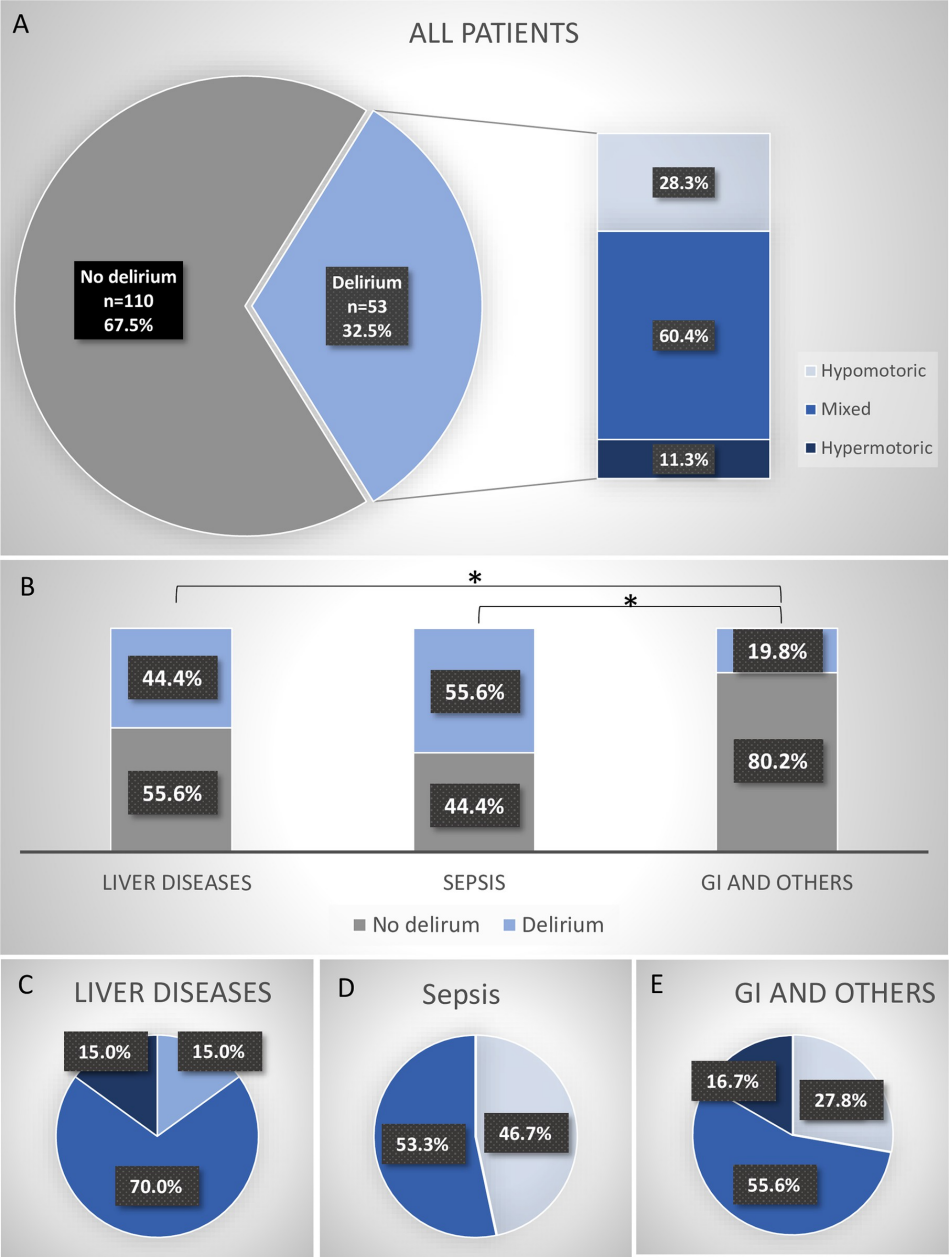
**A:** Incidence and motoric subtypes of delirium. **B:** Liver disease (p = 0,003) and sepsis (p = 0,016) are predictors for delirium. **C:** Motoric subtypes of delirium in patients with liver diseases. **D:** Motoric subtypes of delirium in patients with sepsis. **E:** Motoric subtypes of delirium in patients with GI and other diseases. GI = gastrointestinal.

In our MICU patient cohort, the mixed delirium subtype was predominant with 60.4%, followed by the hypomotoric (28.3%), and hypermotoric (11.3%) subtypes (Fig 3A). A comparison of the incidence of delirium among patients in the three ‘acute medical illness’ groups revealed that patients with liver diseases or sepsis were significantly more likely to develop delirium compared to patients with GI and other diseases (= control group) (44.4% resp. 55.6% vs. 19.8%, p = 0.030 and p = 0.016, respectively) (Fig 3B). Furthermore, there was a trend that patients with liver disease suffered more frequently from the hypermotoric form of delirium than patients with sepsis (15.0% vs. 0%) (Fig 3C and 3D).

### Risk prediction of delirium in the MICU–identification of 10 relevant risk factors

**Fig 4:**
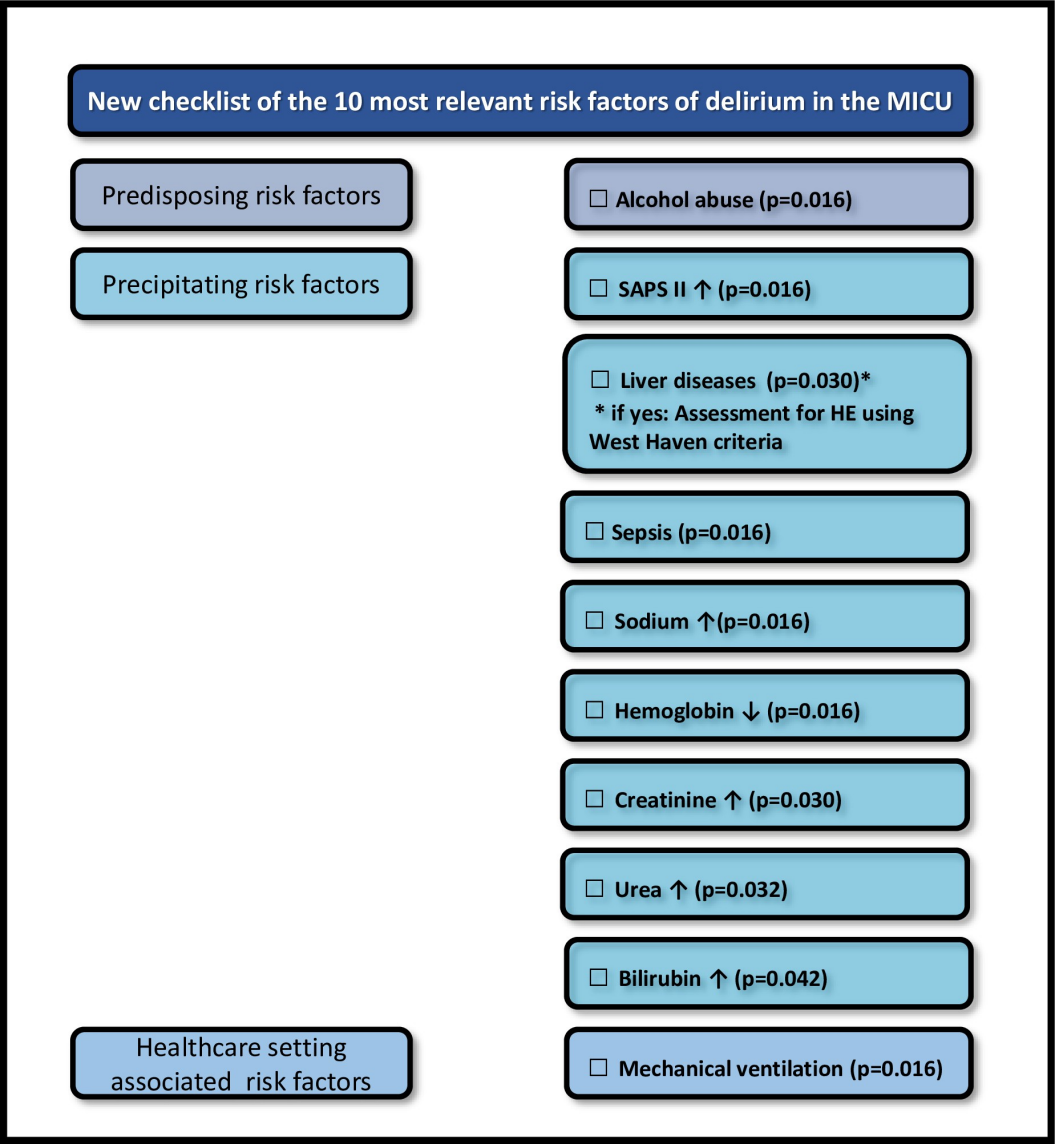
New checklist of the ten clinically most relevant risk factors for delirium in the MICU identified in this study. SAPS II = Simplified Acute Physiology Score, HE = Hepatic Encephalopathy.

Univariable analyses with Holm-Bonferroni correction showed a significant impact of 10 clinically relevant risk factors associated with the development of delirium. These include alcohol abuse (p = 0.016), severity of disease (Simplified Acute Physiology Score II, p = 0.016), liver diseases (p = 0.030) and sepsis (p = 0.016) compared to the control group (gastrointestinal (GI) diseases and others), elevated sodium (p = 0.016), creatinine (p = 0.030), urea (p = 0.032) or bilirubin (p = 0.042), decreased hemoglobin (p = 0.016), and mechanical ventilation (p = 0.016) (Table 4, Fig 4).

**Table 4:** Risk factors of delirium in an MICU.

	Odds ratio	95% CI		p-value after Holm-Bonferroni-correction
**Predisposing risk factors**				
**Age**	1.02	1.00	1.04	0.282
**Sex**	0.65	0.33	1.27	0.830
**BMI**	1.04	0.98	1.10	0.830
**Tobacco use** ^1^	1.56	0.78	3.11	0.830
**Alcohol abuse** ^2^	4.54	2.23	9.24	0.017
**Precipitating risk factors**				
**SAPS II ↑**	1.10	1.06	1.14	0.017
**Acute medical illness** ^3^				
Liver diseases	3.24	1.48	7.09	0.033
Sepsis	5.07	2.02	12.69	0.017
compared to GI diseases and others (= control group)				
**Hepatic encephalopathy** ^4^	3,87	1.09	13.8	0.259
**Sodium ↑**	1.13	1.06	1.20	0.017
**Hemoglobin ↓**	0.78	0.67	0.91	0.017
**Creatinine ↑**	1.73	1.20	2.49	0.033
**Urea ↑**	1.01	1.00	1.02	0.036
**Bilirubin ↑**	1.07	1.02	1.12	0.048
**CRP ↑**	1.00	1.00	1.01	1.000
**Healthcare setting related risk factors**				
**Mechanical ventilation** ^5^	4.81	2.30	10.08	0.017
**Interventions**	0.92	0.43	1.96	1.000

17 univariable binary logistic regression analyses were conducted. Consecutively Bonferroni-Holm p-value adjusting was performed.

^1^reference = no tobacco use

^2^reference = no alcohol abuse

^3^reference = GI diseases and others (control group, n = 91)

^4^only patients with liver diseases were considered

^5^reference = no mechanical ventilation

**↑**/↓ = increase resp. decrease of delirum propability regarding increase in parameter per unit; BMI = Body Mass Index; CI = confidence interval; CRP = C-reactive protein; GI = gastrointestinal; MICU = medical intensive care unit; SAPS II = Simplified Acute Physiology Score.

### Predisposing factors

Predisposing risk factors constitute the background characteristic of patients like age, tobacco use or alcohol abuse [1]. Our studies showed that patients with alcohol abuse as a predisposing factor exhibited a five-time higher delirium probability (95%CI = 2.23–9.24) compared to patients without alcohol abuse (p = 0.016). Increased age was shown to be a predisposing for delirium in univariable analysis (p = 0.047). After Holm-Bonferroni correction this parameter lost its significance. This is most likely due to the age distribution of the patients at this specific MICU with a median of 60 years. The following parameters did not affect delirium probability: age, sex, BMI, and tobacco use.

### Precipitating factors

Precipitating risk factors represent factors associated with the acute medical illness [1]. 45 patients were admitted to the ICU due to liver diseases (group i), 27 patients due to sepsis (group ii) and 91 patients due to GI and other diseases (group iii). The exact diseases classified in the corresponding subgroups are listed in Table 2.

### Liver diseases as a novel risk factor for delirium

In our studies, patients with liver diseases were three times more likely to have delirium (95%CI = 1.48–7.09, p = 0.030) and patients with sepsis were five times more likely to have delirium (95%CI = 2.02–12.39, p = 0.016) compared to patients with GI diseases and other diseases (control group). Patients with liver diseases displayed a comparable and elevated risk of delirium in comparison to patients with sepsis (95%CI = 0.245–1.672, p = 0.361).

### Diagnosis of hepatic encephalopathy based on the West Haven classification in patients with liver diseases

**Fig 5:**
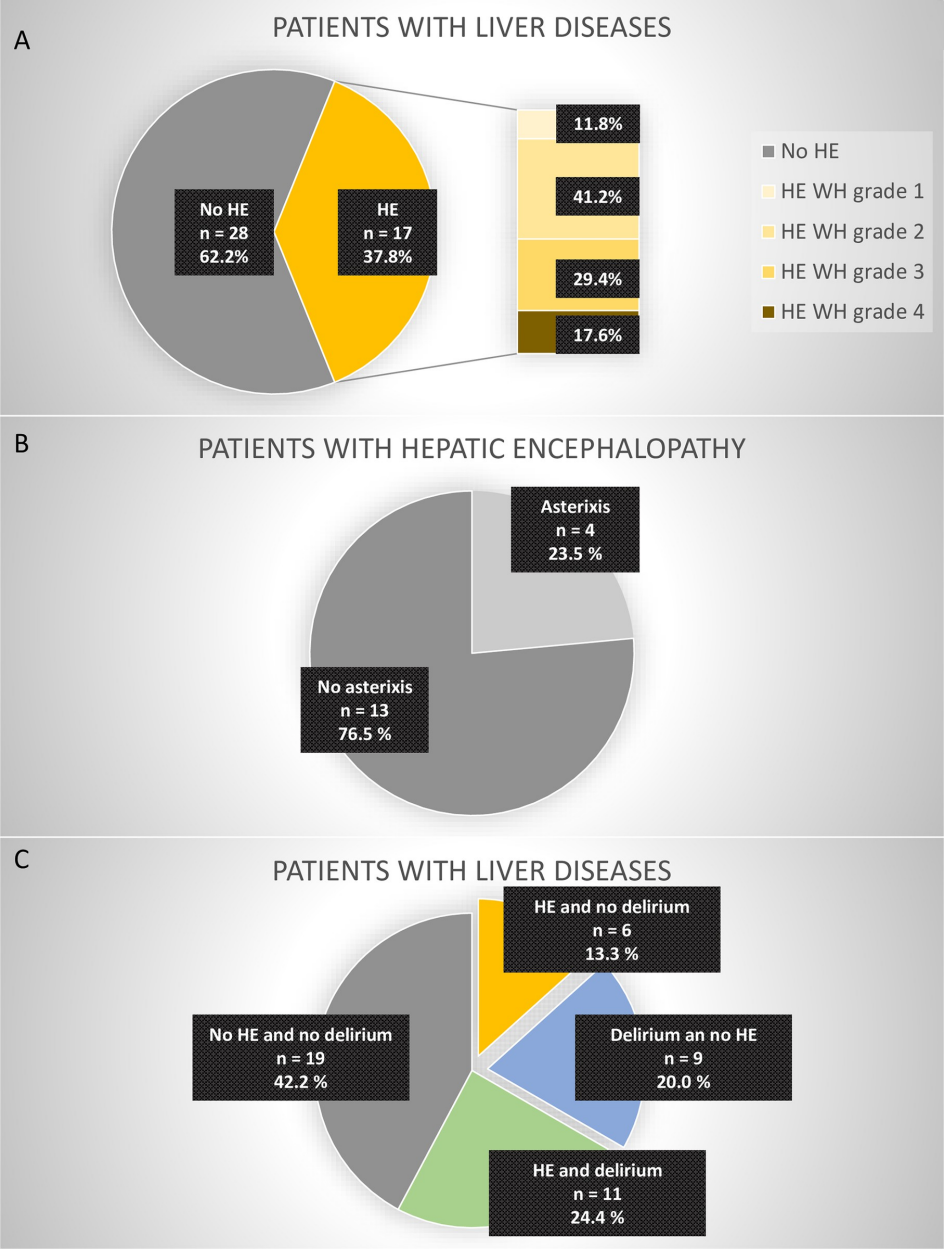
**A:** Hepatic Encephalopathy (HE) classified according to the West Haven (WH) criteria in the subgroup of patients with liver diseases. **B:** asterixis (= flapping hand tremor) in patients with HE. **C:** Incidence of delirium and/or HE in the subgroup of patients with liver disease.

The incidence of HE and/or delirium was analyzed in the subgroup of patients with liver diseases. HE according to the West Haven Criteria was diagnosed in 37.8% of patients with liver diseases, of which 23.5% presented with asterixis (= flapping hand tremor) (Fig 5A and 5B). In 11.8% of patients with hepatic encephalopathy grade 1 HE was diagnosed according to the West Haven Criteria, in 41.2% HE grade 2, in 29.4% HE grade 3 and in 17.6% HE grade 4 (Fig 4). Patients with HE and delirium presented with HE West Haven grade 2.0 (IQR 2.0–3.0) in median, patients with HE and without delirium with a median HE West Haven grade 2.5 (IQR 2.0–4.0) on average. HE was not a risk factor for delirium (p = 0.259). Bilirubin was significantly elevated in patients with hepatic encephalopathy (3.1 mg/dl, IQR = 1.65–17.6) compared to patients without hepatic encephalopathy (0.80 mg/dl, IQR = 0.5–1.8), p<0.001.

While 42.2% of patients with liver diseases had neither HE nor delirium, 13.3% had HE and no delirium, 20.0% had delirium and no HE, and 24.4% had delirium and HE (Fig 5C). In addition, there was no significant difference in West Haven grades between patients with positive or negative CAM-ICU (p = 0.462).

The MELD scores in patients with liver diseases (subgroup i) did not differ significantly between patients with delirium (21.5, IQR = 17.5–28.25) and without delirium (16, IQR = 12.5–26.5), p = 0.102. Thus, the presence of liver disease could per se favor delirium. Of clinical relevance and in accordance with the current literature, the MELD score was shown to be significantly higher in patients with hepatic encephalopathy (26, IQR = 17–30) compared to patients without hepatic encephalopathy (16, IQR = 12–22.75), p = 0.004.

These findings demonstrate that the two entities delirium and HE are independent and should be distinguished in the diagnosis, characterization, and treatment of an altered mental status in the ICU. Delirium and HE may occur concomitantly in some patients with liver diseases, but delirium is not a consequence of HE or vice versa.

### Severity of disease and laboratory parameters as risk factors for delirium

In addition, severity of disease (SAPS II; p = 0.016) was shown to predispose for delirium. Elevated sodium (p = 0.016), creatinine (p = 0.030), urea (p = 0.032), and bilirubin values (p = 0.042) increased the risk of developing delirium. In contrast and of clinical relevance, elevated hemoglobin levels decreased the probability of developing delirium (p = 0.016). The probability of delirium was not affected by C-reactive protein (CRP) (p = 1.000).

### Health-care setting-related factors

Furthermore, health-care setting-related factors for delirium, such as mechanical ventilation and therapeutic interventions were addressed in our studies [1]. After mechanical ventilation, patients showed a five times increased delirium probability (95%CI = 2.18–9.74) compared to patients without previous mechanical ventilation (p = 0.016). 76 interventions, predominantly GI-endoscopies (n = 55, 72.4%), were performed. These interventions did not increase the risk of delirium (p = 1.000).

### SOFA score on admission and at onset of delirium

For all patients the SOFA score at admission was determined. In addition—for patients with delirium—the SOFA Score at onset of delirium was calculated. The SOFA score of all included patients on admission to the ICU showed a median of 5.0 (IQR 2.0–8.0). The median SOFA score for patients with delirium at delirium onset was 6.0 (IQR 4.0–9.0), which was significantly higher than the SOFA Score of all patients on admission (p = 0.008).

### Pharmacological treatment of delirium

The medication patients received during delirium was assessed. Compared to patients without delirium, patients with delirium received significantly more clonidine (p = 0.030), dexmedetomidine (p<0.001), haloperidol (p<0.001) and melperone (p = 0.005) for treatment.

In a subgroup analysis of patients with liver diseases, medication received by patients during delirium was compared to the medication received by patients with HE. This revealed that patients with HE were significantly more likely to be treated with lactulose (p = 0.001).

Regarding the use of haloperidol, it is noteworthy that none of the patients with HE and without delirium were treated with haloperidol. In conclusion, patients with delirium were treated differently in comparison to patients with HE. This is of clinical relevance as it underscores that delirium and HE are two distinct conditions that require specific management and treatment strategies. Making the right diagnosis early on admission to the ICU is crucial in these critically ill patients with altered mental status to initiate an appropriate and specific management plan.

**Fig 6:**
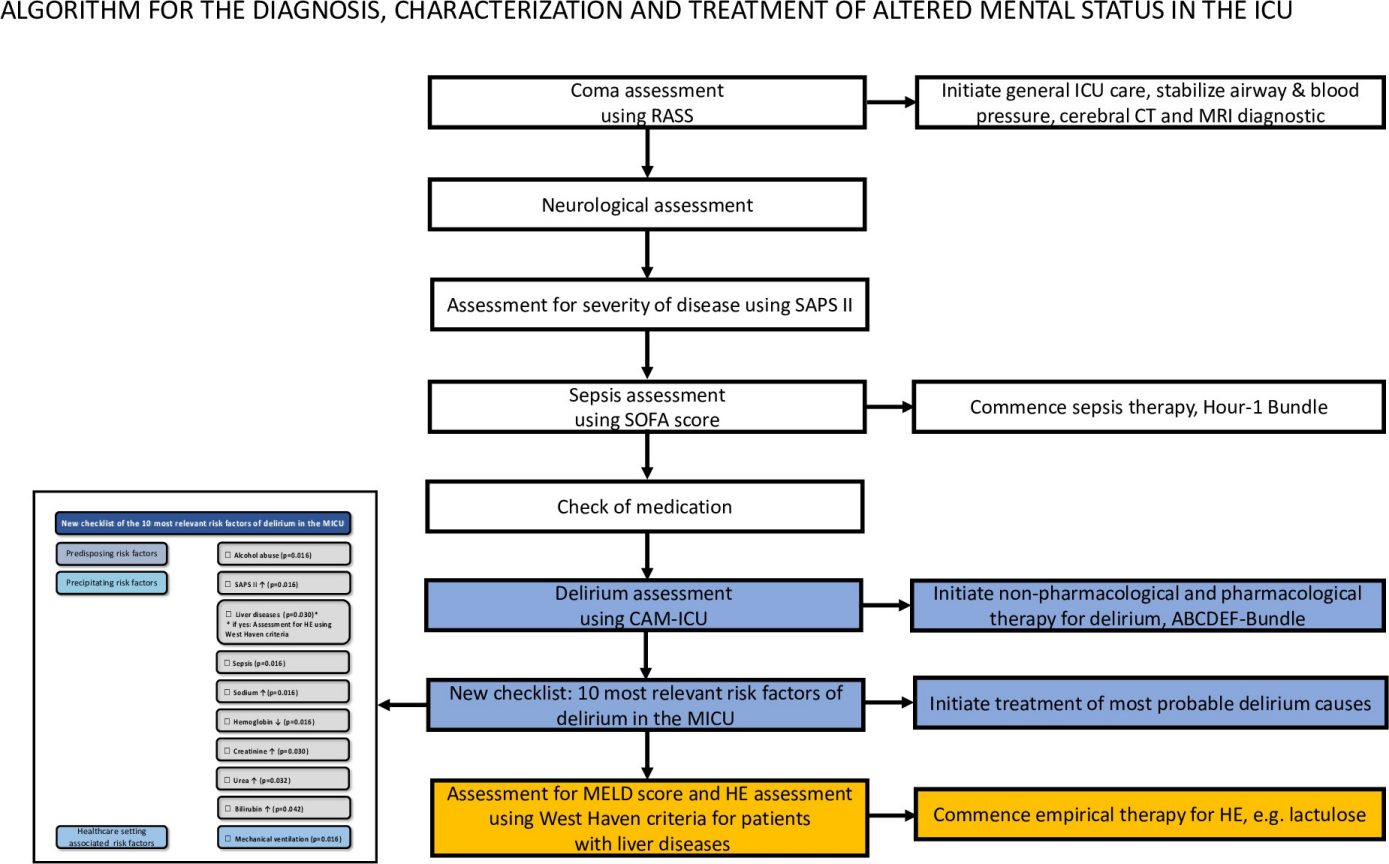
Proposed new algorithm for the diagnosis, characterization and treatment of altered mental status in the ICU.

Therefore, we suggest an algorithm—illustrated in Fig 6 - for the diagnosis, characterization and treatment of altered mental status upon admission to the ICU which includes a new checklist for the 10 most relevant risk factors for delirium and the assessment of HE in patients with liver diseases.

### Clinical outcome of patients with delirium

After identification of the predictors for delirium and assessment of HE in the subgroup of patients with liver diseases, clinical outcome and healthcare system implications were analyzed. Table 5 presents the results of outcome comparisons. Patients with delirium (14 days, IQR = 8.0–20.5) stayed significantly longer in the ICU than patients without delirium (3 days, IQR = 2.0–6.0) (p = 0.004). A total of 20 (12.2%) patients died in-hospital. Patients with delirium were more likely to die in-hospital compared to patients without delirium (22.6 vs. 7.3%, p = 0.036). Patients with delirium were more likely to be transferred to an intensive medical rehabilitation unit as post-intensive care than patients without delirium (19.5% vs. 3.9%, p = 0.02).

**Table 5:** Clinical outcome and healthcare system consequences of patients with delirium.

	Patients without delirium (n = 110)	Patients with delirium (n = 53)	p-value after Holm-Bonferroni-correction
**Clinical outcome**			
**ICU stay** in days (med, IQR)	3.0 (2.0–6.0)	14.0 (8.0–20.5)	0.004
**In-hospital mortality** (n, %)			
No	102 (92.7)	41 (77.4)	0.036
Yes	8 (7.3)	12 (22.6)	
**Post-intensive care** (n, %)^1^			
No	98 (96.1)	33 (79.5)	0.020
Yes	4 (3.9)	8 (19.5)	
**Healthcare system consequences**			
**Healthcare system expenditure** in €	6,020	33,220	0.004
(med, IQR)	(2,954–14,037)	(13,599–61,485)	

Overview of clinical outcome (duration of the ICU stay, transfer to intensive medical rehabilitation unit) and healthcare system consequences (costs in €) of patients with or without delirium.

^1^Transfer to an intensive medical rehabilitation unit after the ICU stay (n = 102 without delirium, n = 41 with delirium).

ICU = intensive care unit; med = Median; IQR = Interquartile range

### Health care expenditure

A calculation of treatment costs by analysis of the G-DRG triggered reimbursement revealed that patients with delirium generated significantly higher treatment costs (p = 0.004). Costs for the healthcare system were 33.220 € (IQR = 13.599 € – 61.485 €) for each patient with delirium vs. 6.020 € (IQR = 2.954 € – 14.037 €) for each patient without delirium (Table 5).

## Discussion

The prevalence of delirium is high in critical care settings. Here, we specifically examined delirium prevalence in an MICU setting in a cohort of patients with liver diseases compared with a patient population with gastrointestinal and other diseases.

53 out of the 163 patients included (32.5%) developed delirium during their MICU stay as diagnosed by the CAM-ICU. This percentage is consistent with the published pooled prevalence of 31.8% in a systemic review of studies from North and South America, Europe and Asia including ventilated and non-ventilated patients [68]. To date, most available data focused on ICUs with a surgical or cardiac focus [2]. Here we provide evidence that the risk of delirium is comparably high in MICU patients with liver diseases and that this study population should be specifically evaluated when screening for delirium to improve the probability of detection, and thus quality of care.

Delirium still remains under-diagnosed, with less than half of delirium cases in hospital being detected. Thus, understanding and reviewing risk factors that predispose to and precipitate delirium is critical for optimal detection, early diagnosis and treatment [1, 39]. Multiple predisposing factors and precipitating factors for delirium have been described. In addition, specific health-care setting-related factors, such as mechanical ventilation are risk factors for delirium.

We have established a new checklist with 10 relevant risk factors for delirium sorted by p value for clinical use in patients referred to the ICU. These include (i) alcohol abuse, (ii) severity of disease (SAPS II), (iii) liver diseases, (iv) sepsis, (v) increased sodium, (vi) decreased hemoglobin, (vii) increased creatinine, (viii) increased urea, (ix) increased bilirubin, and (x) mechanical ventilation. We identified alcohol abuse as a relevant predisposing risk factor for delirium. Chronic alcohol abuse is known to affect the metabolism of the brain due to the influence of GABA-A and NMDA receptors, leading to increased excitability and subsequently to delirium [69]. Age, sex, BMI and tobacco use did not significantly predict delirium in our MICU patient cohort.

That delirium was significantly associated with an increased SAPS II reflects the undisputed fact that severity of disease is an established precipitating risk factor for ICU-acquired delirium. Moreover, delirium per se could also increase the severity of disease [70, 71].

The new findings relate to the diagnosis of an underlying liver disease as an risk factor for delirium. Of note, liver diseases could carry a much higher risk of delirium compared to gastrointestinal or infectious diseases. We therefore suggest that assessment of an underlying liver disease should be included in future delirium prediction models.

Consistent with our recent finding that liver diseases are an important risk factor for delirium, we identified elevated bilirubin levels to be a relevant precipitating factor. High bilirubin concentrations are independent variables associated with the risk of 1-week mortality [72]. A bilirubin concentration ≥3.45 mg/dL in patients with chronic liver disease on hospital admission is a predictor of short-term mortality [73].

Furthermore, the clinical significance of liver disease as precipitating factor for delirium was confirmed in that the statistical significance of risk correlation was comparable to that of patients with sepsis, renal disease and alcohol abuse, which are established risk factors [13, 74, 75].

Of note, HE, which is a well-known complication of liver diseases does not constitute a risk factor for delirium. There was no significant difference in West Haven grades between patients with positive or negative CAM-ICU. The other way round the patients with hepatic encephalopathy had a higher MELD Score than patients without hepatic encephalopathy, but MELD Score between patients with liver disease with and without delirium did not differ. Therefore our results show that HE and delirium are two entities, which should be differentiated.

Therefore, we propose a new algorithm that integrates our data into the respective international guidelines on delirium, sepsis, and liver cirrhosis [62, 76–82]. This algorithm considers the 10 risk factor prediction-model for delirium and prompts grading of the severity of hepatic encephalopathy using the West Haven classification if liver disease is present or newly diagnosed. This diagnostic algorithm could be helpful to distinguish delirium from HE in ICU patients (Fig 6).

We suggest adding “liver disease” as clinically relevant new risk factor when screening for delirium. If the diagnosis of “liver disease” is established, assessment could be completed with the proposed new algorithm for the diagnosis, characterization and treatment of altered mental status, including HE grading according to the West Haven classification.

Hypovolemia is a known risk factor for delirium [4, 43]. Consistently, our study showed that patients with delirium also had significantly elevated sodium levels. However, hemoglobin levels in the normal range appear to reduce the risk of delirium, whereas high sodium levels as a sign of hypovolemia, tend to promote delirium. Therefore, our findings support studies proclaiming that anemia is one of the risk factors for delirium [83, 84]. Of clinical importance, we identified mechanical ventilation as an important health-care setting-related factor for ICU-acquired delirium.

ICU and hospital length of stay were significantly higher for patients with delirium, compared to those without delirium [85–89]. Patients with delirium needed more time to reach a physical and cognitive state which enabled discharge from acute care. In accordance with other published study results [85, 86], our study confirmed that delirium is associated with increased in-hospital mortality [90].

Moreover, patients with delirium generated more than five times higher treatment costs. Only a limited number of studies have focused on the treatment costs of ICU patients with delirium, so far. Weinrebe et al. found that patients with hyperactive delirium generated additional costs of € 1,200 [87]. In our MICU cohort, the mean difference in DRG-based reimbursement for patients with and without delirium was considerably higher € 27,199 (€ 6,021 vs. € 33,220). This considerably larger difference in treatment costs may be explained by the fact that, in contrast to the study by Weinrebe et al. [87], our study was conducted in an ICU. Furthermore, we also integrated patients with hypoactive and mixed subtype of delirium in our analyses.

Regarding transfer goals, we found that patients with delirium were five times more likely to be transferred to an intensive medical rehabilitation unit for post-intensive care. This finding may reflect the protracted course of recovery following delirium and or hepatic encephalopathy of patients with chronic liver disease [88, 89].

### Limitations

This study has several limitations. First, the sample size is small. Second, this study is a single-center study. Larger-scale multi-center studies are needed to confirm these findings, especially to confirm the detected risk factors. Another limitation is that the treatment costs were calculated according to the G-DRG triggered reimbursement, treatment costs essentially reflect the situation in Europe.

## Conclusions

In summary, delirium is of high relevance in an MICU with focus on liver, gastrointestinal and infectious diseases. Particular attention should be paid to patients with liver disease to enable prevention, early identification, and intervention in this patient cohort. It is of therapeutical relevance to clearly differentiate delirium and hepatic encephalopathy in this population. Based on our data, “liver diseases” should be considered as a clinically relevant new risk factor when screening for delirium. Therefore, we propose a new algorithm that integrates our data into the respective international guidelines on delirium, sepsis, and liver cirrhosis.

## List of abbreviations

ACLF: Acute-on-chronic liver failure
BMI: Body mass index
CAM: Confusion Assessment Method
CI: Confidence interval
CRP: C-reactive protein
DRG: Diagnosis-related Groups
DSM: Diagnostic and Statistical Manual of Mental Disorders
GABA-A: γ-Aminobutyric acid type A
G-DRG: German Diagnosis-related Groups
GI: Gastrointestinal
h: Hours
HE: Hepatic Encephalopathy
ICU: Intensive care unit
IQR: Interquartile range
med: Median
MICU: Medical intensive care unit
NMDA: N-methyl-D-aspartate
OR: Odds ratio
PCT: Procalcitonin
PSC: Primary sclerosing cholangitis
RASS: Richmond Agitation Sedation Scale
SAPS: Simplified Acute Physiology Score
SOFA score: sequential organ failure assessment score
WH: West Haven

## References

[1] Wilson JE, Mart MF, Cunningham C, Shehabi Y, Girard TD, MacLullich AMJ, et al. Delirium. Nat Rev Dis Prim [Internet]. 2020 Dec;6(1):90. Available from: http://www.nature.com/articles/s41572-020-00223-4 33184265 10.1038/s41572-020-00223-4 PMC9012267

[2] Pandharipande PP, Ely EW, Arora RC, Balas MC, Boustani MA, La Calle GH, et al. The intensive care delirium research agenda: a multinational, interprofessional perspective. Intensive Care Med [Internet]. 2017 Sep;43(9):1329–39. Available from: http://link.springer.com/10.1007/s00134-017-4860-7 28612089 10.1007/s00134-017-4860-7 PMC5709210

[3] Barr J, Fraser GL, Puntillo K, Ely EW, Gélinas C, Dasta JF, et al. Clinical Practice Guidelines for the Management of Pain, Agitation, and Delirium in Adult Patients in the Intensive Care Unit. Crit Care Med [Internet]. 2013 Jan;41(1):263–306. Available from: https://insights.ovid.com/crossref?an=00003246-201301000-00029 doi: 10.1097/CCM.0b013e3182783b72 23269131

[4] Gibb K, Seeley A, Quinn T, Siddiqi N, Shenkin S, Rockwood K, et al. The consistent burden in published estimates of delirium occurrence in medical inpatients over four decades: A systematic review and meta-analysis study. Age and Ageing. 2020. doi: 10.1093/ageing/afaa040 32239173 PMC7187871

[5] Stollings JL, Kotfis K, Chanques G, Pun BT, Pandharipande PP, Ely EW. Delirium in critical illness: clinical manifestations, outcomes, and management. Intensive Care Medicine. 2021. 10.1007/s00134-021-06503-1 PMC8366492 34401939

[6] American Psychiatric Association. Diagnostic and Statistical Manual of Mental Disorders [Internet]. American Psychiatric Association; 2013. Available from: https://psychiatryonline.org/doi/book/10.1176/appi.books.9780890425596

[7] European Delirium Association* and American Delirium Society. The DSM-5 criteria, level of arousal and delirium diagnosis: inclusiveness is safer. Available from: https://bmcmedicine.biomedcentral.com/track/pdf/10.1186/s12916-014-0141-2?site=bmcmedicine.biomedcentral.com 10.1186/s12916-014-0141-2 PMC4177077 25300023

[8] Slooter AJC, Otte WM, Devlin JW, Arora RC, Bleck TP, Claassen J, et al. Updated nomenclature of delirium and acute encephalopathy: statement of ten Societies. Intensive Care Med [Internet]. 2020 May;46(5):1020–2. Available from: http://link.springer.com/10.1007/s00134-019-05907-4 32055887 10.1007/s00134-019-05907-4 PMC7210231

[9] Sessler CN, Gosnell MS, Grap MJ, Brophy GM, O’Neal P V, Keane KA, et al. The Richmond Agitation–Sedation Scale. Am J Respir Crit Care Med [Internet]. 2002 Nov;166(10):1338–44. Available from: http://www.ncbi.nlm.nih.gov/pubmed/12421743 12421743 10.1164/rccm.2107138

[10] Peterson JF, Pun BT, Dittus RS, Thomason JWW, Jackson JC, Ayumi Ã, et al. Delirium and Its Motoric Subtypes: A Study of 614 Critically Ill Patients. J Am Geriatr Soc [Internet]. 2006;54:479–84. Available from: https://www.researchgate.net/profile/James_Jackson7/publication/7228300_Delirium_and_Its_Motoric_Subtypes_A_Study_of_614_Critically_Ill_Patients/links/5405ea480cf2bba34c1def92/Delirium-and-Its-Motoric-Subtypes-A-Study-of-614-Critically-Ill-Patients.pdf doi: 10.1111/j.1532-5415.2005.00621.x 16551316

[11] Mcnicoll L, Pisani MA, Zhang Y, Ely EW, Siegel MD, Inouye SK. Delirium in the Intensive Care Unit: Occurrence and Clinical Course in Older Patients. JAGS [Internet]. 2003;51:591–8. Available from: https://pdfs.semanticscholar.org/c72a/7d35fd472d9d83b034017cde02b948bf7f38.pdf doi: 10.1034/j.1600-0579.2003.00201.x 12752832

[12] la Cour KN, Andersen-Ranberg NC, Weihe S, Poulsen LM, Mortensen CB, Kjer CKW, et al. Distribution of delirium motor subtypes in the intensive care unit: a systematic scoping review. Crit Care. 2022; doi: 10.1186/s13054-022-03931-3 35241132 PMC8896322

[13] Maldonado JR. Delirium pathophysiology: An updated hypothesis of the etiology of acute brain failure. In: International Journal of Geriatric Psychiatry. 2018. doi: 10.1002/gps.4823 29278283

[14] Maschke M. Delir und Verwirrtheitszustände inklusive Alkoholentzugsdelir Entwicklungsstufe: S1 Leitlinien für Diagnostik und Therapie in der Neurologie [Internet]. 2020. Available from: www.awmf.org

[15] Adelman MW, Woodworth MH, Langelier C, Busch LM, Kempker JA, Kraft CS, et al. The gut microbiome’s role in the development, maintenance, and outcomes of sepsis. Crit Care [Internet]. 2020;24(1):278. Available from: http://www.ncbi.nlm.nih.gov/pubmed/32487252 doi: 10.1186/s13054-020-02989-1 32487252 PMC7266132

[16] Haller A. Das Delir auf der Intensivstation. Available from: https://medicalforum.ch/de/resource/jf/journal/file/view/article/smf.2015.02351/smf-02351.pdf/

[17] Francis J, Young GB. Diagnosis of delirium and confusional states. UpToDate [Internet]. 2012;1–20. Available from: https://www.uptodate.com/contents/diagnosis-of-delirium-and-confusional-states?source=search_result&search=delir&selectedTitle=1%7B~%7D150

[18] Kudoh A, Takase H, Katagai H, Takazawa T. Postoperative Interleukin-6 and Cortisol Concentrations in Elderly Patients with Postoperative Confusion. Neuroimmunomodulation [Internet]. 2005;12:60–6. Available from: www.karger.com doi: 10.1159/000082365 15756054

[19] O’Keeffe ST, Devlin JG. Delirium and the Dexamethasone Suppression Test in the Elderly. Neuropsychobiology [Internet]. 1994;30(4):153–6. Available from: http://www.ncbi.nlm.nih.gov/pubmed/7862262 doi: 10.1159/000119154 7862262

[20] van Gool WA, van de Beek D, Eikelenboom P. Systemic infection and delirium: when cytokines and acetylcholine collide. The Lancet. 2010. doi: 10.1016/S0140-6736(09)61158-2 20189029

[21] van den Boogaard M, Kox M, Quinn KL, van Achterberg T, van der Hoeven JG, Schoonhoven L, et al. Biomarkers associated with delirium in critically ill patients and their relation with long-term subjective cognitive dysfunction; indications for different pathways governing delirium in inflamed and noninflamed patients. Crit Care. 2011; doi: 10.1186/cc10598 22206727 PMC3388649

[22] Caplan GA, Kvelde T, Lai C, Yap SL, Lin C, Hill MA. Cerebrospinal Fluid in Long-Lasting Delirium Compared With Alzheimer’s Dementia. Journals Gerontol Ser A Biol Sci Med Sci [Internet]. 2010 Oct;65A(10):1130–6. Available from: https://academic.oup.com/biomedgerontology/article-lookup/doi/10.1093/gerona/glq090 20530241 10.1093/gerona/glq090

[23] Schoen J, Meyerrose J, Paarmann H, Heringlake M, Hueppe M, Berger K-U. Preoperative regional cerebral oxygen saturation is a predictor of postoperative delirium in on-pump cardiac surgery patients: a prospective observational trial. Crit Care [Internet]. 2011;15(5):R218. Available from: http://www.ncbi.nlm.nih.gov/pubmed/21929765 doi: 10.1186/cc10454 21929765 PMC3334763

[24] Kochunov P, Ramage AE, Lancaster JL, Robin DA, Narayana S, Coyle T, et al. Loss of cerebral white matter structural integrity tracks the gray matter metabolic decline in normal aging. Neuroimage [Internet]. 2009 Mar;45(1):17–28. Available from: http://www.ncbi.nlm.nih.gov/pubmed/19095067 doi: 10.1016/j.neuroimage.2008.11.010 19095067 PMC2734283

[25] Coleman PD, Flood DG. Neuron numbers and dendritic extent in normal aging and Alzheimer’s disease. Neurobiol Aging [Internet]. 1987 Nov;8(6):521–45. Available from: https://linkinghub.elsevier.com/retrieve/pii/0197458087901278 doi: 10.1016/0197-4580(87)90127-8 3323927

[26] Kazmierski J, Banys A, Latek J, Bourke J, Jaszewski R. Cortisol levels and neuropsychiatric diagnosis as markers of postoperative delirium: a prospective cohort study [Internet]. Vol. 17, Critical Care. 2013. Available from: http://ccforum.com/content/17/2/R38 10.1186/cc12548 PMC3733427 23452669

[27] Juraska JM, Lowry NC. Neuroanatomical Changes Associated with Cognitive Aging. In Springer, Berlin, Heidelberg; 2011. p. 137–62. Available from: http://link.springer.com/10.1007/7854_2011_137 10.1007/7854_2011_137 21671190

[28] Kelly KM, Nadon NL, Morrison JH, Thibault O, Barnes CA, Blalock EM. The neurobiology of aging. Epilepsy Res [Internet]. 2006;68:5–20. Available from: http://www.nia.nih.gov/research/resources.htm doi: 10.1016/j.eplepsyres.2005.07.015 16386406

[29] Balan S, Leibovitz A, Zila SO, Ruth M, Chana W, Yassica B, et al. The Relation Between the Clinical Subtypes of Delirium and the Urinary Level of 6-SMT. J Neuropsychiatry Clin Neurosci [Internet]. 2003 Aug;15(3):363–6. Available from: http://www.ncbi.nlm.nih.gov/pubmed/12928514 doi: 10.1176/jnp.15.3.363 12928514

[30] Haron S, Nouye KI, Idney S, Ogardus TB, Harpentier EAC, Inda L, et al. A multicomponent intervention to prevent delirium in hospitalized older patients Background Since in hospitalized older patients. 1999;340(9). Available from: http://www.nejm.org/doi/pdf/10.1056/NEJM199903043400901 10.1056/NEJM199903043400901 10053175

[31] Schiemann A, Hadzidiakos D, Spies C. Managing ICU delirium. Curr Opin Crit Care [Internet]. 2011 Apr;17(2):131–40. Available from: http://www.ncbi.nlm.nih.gov/pubmed/21301333 doi: 10.1097/MCC.0b013e32834400b5 21301333

[32] Witlox J, Eurelings LSM, de Jonghe JFM, Kalisvaart KJ, Eikelenboom P, van Gool WA. Delirium in elderly patients and the risk of postdischarge mortality, institutionalization, and dementia: a meta-analysis. JAMA [Internet]. 2010 Jul;304(4):443–51. Available from: http://www.ncbi.nlm.nih.gov/pubmed/20664045 doi: 10.1001/jama.2010.1013 20664045

[33] Sanchez D, Brennan K, Al Sayfe M, Shunker S-A, Bogdanoski T, Hedges S, et al. Frailty, delirium and hospital mortality of older adults admitted to intensive care: the Delirium (Deli) in ICU study. Crit Care [Internet]. 2020;24(1):609. Available from: http://www.ncbi.nlm.nih.gov/pubmed/33059749 doi: 10.1186/s13054-020-03318-2 33059749 PMC7565834

[34] Marcantonio ER. Delirium in Hospitalized Older Adults. Solomon CG, editor. N Engl J Med [Internet]. 2017 Oct;377(15):1456–66. Available from: http://www.nejm.org/doi/10.1056/NEJMcp1605501 29020579 10.1056/NEJMcp1605501 PMC5706782

[35] Inouye SK, Westendorp RGJ, Saczynski JS. Delirium in elderly people. Lancet [Internet]. 2014 Mar;383(9920):911–22. Available from: https://www.sciencedirect.com/science/article/pii/S0140673613606881 doi: 10.1016/S0140-6736(13)60688-1 23992774 PMC4120864

[36] van den Boogaard M, Pickkers P, Slooter AJC, Kuiper MA, Spronk PE, van der Voort PHJ, et al. Development and validation of PRE-DELIRIC (PREdiction of DELIRium in ICu patients) delirium prediction model for intensive care patients: observational multicentre study. BMJ [Internet]. 2012 Feb;344:e420. Available from: http://www.ncbi.nlm.nih.gov/pubmed/22323509 doi: 10.1136/bmj.e420 22323509 PMC3276486

[37] Palakshappa JA, Hough CL. How We Prevent and Treat Delirium in the ICU. Chest. 2021. doi: 10.1016/j.chest.2021.06.002 34102141 PMC8727852

[38] Aung Thein MZ, Pereira J V, Nitchingham A, Caplan GA. A call to action for delirium research: Meta-analysis and regression of delirium associated mortality. BMC Geriatr [Internet]. 2020 Dec;20(1):325. Available from: https://bmcgeriatr.biomedcentral.com/articles/10.1186/s12877-020-01723-4 32894065 10.1186/s12877-020-01723-4 PMC7487610

[39] Welch C, McCluskey L, Wilson D, Chapman GE, Jackson TA, Treml J, et al. Delirium is prevalent in older hospital inpatients and associated with adverse outcomes: Results of a prospective multi-centre study on World Delirium Awareness Day. BMC Med. 2019. 10.1186/s12916-019-1458-7 PMC6911703 31837711

[40] Moraes FDS, Marengo LL, Silva MT, De Cassia Bergamaschi C, Lopes LC, Del Grossi Moura M, et al. ABCDE and ABCDEF care bundles: A systematic review protocol of the implementation process in intensive care units. Medicine (United States). 2019. doi: 10.1097/MD.0000000000014792 30882653 PMC6426482

[41] Burton JK, Craig LE, Yong SQ, Siddiqi N, Teale EA, Woodhouse R, et al. Non-pharmacological interventions for preventing delirium in hospitalised non-ICU patients. Cochrane Database of Systematic Reviews. 2021. 10.1002/14651858.CD013307.pub2 PMC8407051 34280303

[42] Gu W, Hortlik H, Erasmus HP, Schaaf L, Zeleke Y, Uschner FE, et al. Trends and the course of liver cirrhosis and its complications in Germany: Nationwide population-based study (2005 to 2018). Lancet Reg Heal—Eur. 2022. doi: 10.1016/j.lanepe.2021.100240 34901909 PMC8640738

[43] Bajaj JS, Gentili A, Wade JB, Godschalk M. Specific Challenges in Geriatric Cirrhosis and Hepatic Encephalopathy. Clin Gastroenterol Hepatol [Internet]. 2022;20(8, Supplement):S20–9. Available from: https://www.sciencedirect.com/science/article/pii/S1542356522004633 doi: 10.1016/j.cgh.2022.04.035 35940730 PMC9373233

[44] Mezzano G, Juanola A, Cardenas A, Mezey E, Hamilton JP, Pose E, et al. Global burden of disease: Acute-on-chronic liver failure, a systematic review and meta-analysis. Gut. 2022;71(1). doi: 10.1136/gutjnl-2020-322161 33436495

[45] Nanchal R, Subramanian R, Karvellas CJ, Hollenberg SM, Peppard WJ, Singbartl K, et al. Guidelines for the management of adult acute and acute-on-chronic liver failure in the ICU: Cardiovascular, endocrine, hematologic, pulmonary and renal considerations: Executive summary. Crit Care Med. 2020. doi: 10.1097/CCM.0000000000004193 32058375

[46] Bajaj J, O’Leary J, Lai J, Wong F, Long M, Wong R, et al. Acute-on-Chronic Liver Failure Clinical Guidelines. Am J Gastroenterol. 2022;117(2):225–52. doi: 10.14309/ajg.0000000000001595 35006099

[47] Angeli P, Bernardi M, Villanueva C, Francoz C, Mookerjee RP, Trebicka J, et al. EASL Clinical Practice Guidelines for the management of patients with decompensated cirrhosis. J Hepatol. 2018. 10.1016/j.jhep.2018.03.024 29653741

[48] Sarin SK, Choudhury A, Sharma MK, Maiwall R, Al Mahtab M, Rahman S, et al. Acute-on-chronic liver failure: consensus recommendations of the Asian Pacific association for the study of the liver (APASL): an update. Hepatol Int. 2019;13(4). 10.1007/s12072-019-09946-3 PMC6728300 31172417

[49] Meersseman P, Langouche L, du Plessis J, Korf H, Mekeirele M, Laleman W, et al. The intensive care unit course and outcome in acute-on-chronic liver failure are comparable to other populations. J Hepatol. 2018;69(4). doi: 10.1016/j.jhep.2018.04.025 29730473

[50] American Psychiatric Association Diagnostic and Statistical Manual of Mental Disorders (DSM-IV). In: SpringerReference. 2012.

[51] Gusmao-Flores D, Salluh JIF, Chalhub RÁ, Quarantini LC. The confusion assessment method for the intensive care unit (CAM-ICU) and intensive care delirium screening checklist (ICDSC) for the diagnosis of delirium: a systematic review and meta-analysis of clinical studies. Crit Care [Internet]. 2012 Jul;16(4):R115. Available from: http://www.ncbi.nlm.nih.gov/pubmed/22759376 doi: 10.1186/cc11407 22759376 PMC3580690

[52] Inouye SK, Van Dyck CH, Alessi CA, Balkin S, Siegal AP, Horwitz RI. Clarifying confusion: The confusion assessment method: A new method for detection of delirium. Ann Intern Med. 1990. 10.7326/0003-4819-113-12-941 2240918

[53] Shenkin SD, Fox C, Godfrey M, Siddiqi N, Goodacre S, Young J, et al. Delirium detection in older acute medical inpatients: A multicentre prospective comparative diagnostic test accuracy study of the 4AT and the confusion assessment method. BMC Med. 2019. doi: 10.1186/s12916-019-1367-9 31337404 PMC6651960

[54] Burger M, Brönstrup A, Pietrzik K. Derivation of tolerable upper alcohol intake levels in Germany: a systematic review of risks and benefits of moderate alcohol consumption. Prev Med (Baltim) [Internet]. 2004 Jul;39(1):111–27. Available from: https://www.sciencedirect.com/science/article/pii/S0091743503003256 doi: 10.1016/j.ypmed.2003.11.011 15207992

[55] Vilstrup H, Amodio P, Bajaj J, Cordoba J, Ferenci P, Mullen KD, et al. Hepatic encephalopathy in chronic liver disease: 2014 Practice Guideline by the American Association for the Study Of Liver Diseases and the European Association for the Study of the Liver. Hepatology. 2014;60(2). 10.1002/hep.27210 25042402

[56] Bajaj JS, Cordoba J, Mullen KD, Amodio P, Shawcross DL, Butterworth RF, et al. Review article: The design of clinical trials in hepatic encephalopathy—An International Society for Hepatic Encephalopathy and Nitrogen Metabolism (ISHEN) consensus statement. Vol. 33, Alimentary Pharmacology and Therapeutics. 2011. 10.1111/j.1365-2036.2011.04590.x PMC3971432 21306407

[57] Montagnese S, Amodio P, Morgan MY. Methods for diagnosing hepatic encephalopathy in patients with cirrhosis: A multidimensional approach. In: Metabolic Brain Disease. 2004. doi: 10.1023/b:mebr.0000043977.11113.2a 15554423

[58] Kamath P, Wiesner R, Malinchoc M, Kremers W, Therneau T, Damico G, et al. A model to predict survival in patients with end-stage liver disease. Gastroenterology. 2001. 10.1053/jhep.2001.22172 11172350

[59] Kamath PS, Kim WR. The Model for End-stage Liver Disease (MELD). Hepatology. 2007. 10.1002/hep.21563 17326206

[60] Seymour CW, Liu VX, Iwashyna TJ, Brunkhorst FM, Rea TD, Scherag A, et al. Assessment of clinical criteria for sepsis for the third international consensus definitions for sepsis and septic shock (sepsis-3). JAMA—J Am Med Assoc. 2016;315(8):762–74. 10.1001/jama.2016.0288 PMC5433435 26903335

[61] Lopes Ferreira F, Peres Bota D, Bross A, Mélot C, Vincent JL. Serial evaluation of the SOFA score to predict outcome in critically ill patients. J Am Med Assoc. 2001;286(14). 10.1001/jama.286.14.1754 11594901

[62] Singer M, Deutschman CS, Seymour C, Shankar-Hari M, Annane D, Bauer M, et al. The third international consensus definitions for sepsis and septic shock (sepsis-3). Vol. 315, JAMA—Journal of the American Medical Association. 2016. p. 801–10. 10.1001/jama.2016.0287 PMC4968574 26903338

[63] Shankar-Hari M, Phillips GS, Levy ML, Seymour CW, Liu VX, Deutschman CS, et al. Developing a newdefinition and assessing newclinical criteria for Septic shock: For the third international consensus definitions for sepsis and septic shock (sepsis-3). JAMA—J Am Med Assoc. 2016;315(8).

[64] Lambden S, Laterre PF, Levy MM, Francois B. The SOFA score—Development, utility and challenges of accurate assessment in clinical trials. Vol. 23, Critical Care. 2019. doi: 10.1186/s13054-019-2663-7 31775846 PMC6880479

[65] Sendagire C, Lipnick MS, Kizito S, Kruisselbrink R, Obua D, Ejoku J, et al. Feasibility of the modified sequential organ function assessment score in a resource-constrained setting: A prospective observational study. BMC Anesthesiol. 2017;17(1). 10.1186/s12871-017-0304-8 PMC5267406 28122489

[66] Vincent JL, Rello J, Marshall J, Silva E, Anzueto A, Martin CD, et al. International study of the prevalence and outcomes of infection in intensive care units. JAMA—J Am Med Assoc. 2009;302(21). 10.1001/jama.2009.1754 19952319

[67] Siddiqi N, Harrison JK, Clegg A, Teale EA, Young J, Taylor J, et al. Interventions for preventing delirium in hospitalised non-ICU patients. Cochrane database Syst Rev [Internet]. 2016 Mar;3:CD005563. Available from: http://www.ncbi.nlm.nih.gov/pubmed/26967259 doi: 10.1002/14651858.CD005563.pub3 26967259 PMC10431752

[68] Krewulak KD, Stelfox HT, Leigh JP, Ely EW, Fiest KM. Incidence and Prevalence of Delirium Subtypes in an Adult ICU: A Systematic Review and Meta-Analysis. Crit Care Med [Internet]. 2018;46(12):2029–35. Available from: http://www.ncbi.nlm.nih.gov/pubmed/30234569 doi: 10.1097/CCM.0000000000003402 30234569

[69] Sarkar S, Choudhury S, Ezhumalai G, Konthoujam J. Risk factors for the development of delirium in alcohol dependence syndrome: Clinical and neurobiological implications. Indian J Psychiatry [Internet]. 2017;59(3):300–5. Available from: http://www.ncbi.nlm.nih.gov/pubmed/29085088 doi: 10.4103/psychiatry.IndianJPsychiatry_67_17 29085088 PMC5659079

[70] Abelha FJ, Fernandes V, Botelho M, Santos P, Santos A, Machado JC, et al. Apolipoprotein E e4 allele does not increase the risk of early postoperative delirium after major surgery. J Anesth [Internet]. 2012 Feb; Available from: http://www.ncbi.nlm.nih.gov/pubmed/22302107 10.1007/s00540-012-1326-5 22302107

[71] Estrup S, Kjer CKW, Poulsen LM, Gøgenur I, Mathiesen O. Delirium and effect of circadian light in the intensive care unit: a retrospective cohort study. Acta Anaesthesiol Scand [Internet]. 2018 Mar;62(3):367–75. Available from: http://www.ncbi.nlm.nih.gov/pubmed/29148046 doi: 10.1111/aas.13037 29148046

[72] López-Velázquez JA, Chávez-Tapia NC, Ponciano-Rodríguez G, Sánchez-Valle V, Caldwell SH, Uribe M, et al. Bilirubin alone as a biomarker for short-term mortality in acute-on-chronic liver failure: An important prognostic indicator. Ann Hepatol. 2014;13(1). 24378272

[73] Méndez-Sánchez N, Vítek L, Aguilar-Olivos NE, Uribe M. Bilirubin as a Biomarker in Liver Disease. In 2017.

[74] Siew ED, Fissell WH, Tripp CM, Blume JD, Wilson MD, Clark AJ, et al. Acute Kidney Injury as a Risk Factor for Delirium and Coma during Critical Illness. Am J Respir Crit Care Med [Internet]. 2017 Jun;195(12):1597–607. Available from: http://www.atsjournals.org/doi/10.1164/rccm.201603-0476OC 27854517 10.1164/rccm.201603-0476OC PMC5476907

[75] Pisani MA, Murphy TE, Van Ness PH, Araujo KLB, Inouye SK. Characteristics associated with delirium in older patients in a medical intensive care unit. Arch Intern Med [Internet]. 2007;167(15):1629–34. Available from: http://www.ncbi.nlm.nih.gov/pubmed/17698685 doi: 10.1001/archinte.167.15.1629 17698685

[76] Devlin JW, Skrobik Y, Gélinas C, Needham DM, Slooter AJC, Pandharipande PP, et al. Clinical Practice Guidelines for the Prevention and Management of Pain, Agitation/Sedation, Delirium, Immobility, and Sleep Disruption in Adult Patients in the ICU. Crit Care Med. 2018; 10.1097/CCM.0000000000003299 30113379

[77] Maschke M. S1-Leitlinie: Delir und Verwirrtheitszustände inklusive Alkoholentzugsdelir. DGNeurologie. 2021.

[78] Barr J, Fraser GL, Puntillo K, Ely EW, Gélinas C, Dasta JF, et al. Clinical practice guidelines for the management of pain, agitation, and delirium in adult patients in the intensive care unit: Executive summary. American Journal of Health-System Pharmacy. 2013. 10.1093/ajhp/70.1.53 23261901

[79] Aldecoa C, Bettelli G, Bilotta F, Sanders RD, Audisio R, Borozdina A, et al. European Society of Anaesthesiology evidence-based and consensus-based guideline on postoperative delirium. Eur J Anaesthesiol. 2017. doi: 10.1097/EJA.0000000000000594 28187050

[80] Lammert F, Acalovschi M, Ercolani G, van Erpecum KJ, Gurusamy KS, van Laarhoven CJ, et al. EASL Clinical Practice Guidelines on the prevention, diagnosis and treatment of gallstones. J Hepatol. 2016. 10.1016/j.jhep.2016.03.005 27085810

[81] Rogal SS, Hansen L, Patel A, Ufere NN, Verma M, Woodrell CD, et al. AASLD Practice Guidance: Palliative care and symptom-based management in decompensated cirrhosis. Hepatology [Internet]. 2022;76(3):819–53. Available from: https://aasldpubs.onlinelibrary.wiley.com/doi/abs/10.1002/hep.32378 35103995 10.1002/hep.32378 PMC9942270

[82] Gerbes AL, Labenz J, Appenrodt B, Dollinger M, Gundling F, Gülberg V, et al. Aktualisierte S2k-Leitlinie der Deutschen Gesellschaft für Gastroenterologie, Verdauungs- und Stoffwechselkrankheiten (DGVS) „Komplikationen der Leberzirrhose“. Z Gastroenterol. 2019. 10.1055/a-0873-4658 31060080

[83] Joosten E, Lemiengre J, Nelis T, Verbeke G, Milisen K. Is Anaemia a Risk Factor for Delirium in an Acute Geriatric Population? Gerontology [Internet]. 2006;52(6):382–5. Available from: http://www.ncbi.nlm.nih.gov/pubmed/16914932 doi: 10.1159/000095126 16914932

[84] Granberg Axell AIR, Malmros CW, Bergbom IL, Lundberg DBA. Intensive care unit syndrome/delirium is associated with anemia, drug therapy and duration of ventilation treatment. Acta Anaesthesiol Scand [Internet]. 2002 Jul;46(6):726–31. Available from: http://doi.wiley.com/10.1034/j.1399-6576.2002.460616.x 12059899 10.1034/j.1399-6576.2002.460616.x

[85] Salluh JIF, Wang H, Schneider EB, Nagaraja N, Yenokyan G, Damluji A, et al. Outcome of delirium in critically ill patients: systematic review and meta-analysis. Bmj [Internet]. 2015;350(may19 3):h2538—h2538. Available from: http://www.bmj.com/cgi/doi/10.1136/bmj.h2538 26041151 10.1136/bmj.h2538 PMC4454920

[86] Cole M, McCusker J, Dendukuri N, Han L. The Prognostic Significance of Subsyndromal Delirium in Elderly Medical Inpatients. J Am Geriatr Soc [Internet]. 2003 Jun;51(6):754–60. Available from: http://doi.wiley.com/10.1046/j.1365-2389.2003.51255.x 12757560 10.1046/j.1365-2389.2003.51255.x

[87] Weinrebe W, Johannsdottir E, Karaman M, Füsgen I. What does delirium cost? An economic evaluation of hyperactive delirium. Z Gerontol Geriatr [Internet]. 2016 Jan;49(1):52–8. Available from: http://www.ncbi.nlm.nih.gov/pubmed/25801513 doi: 10.1007/s00391-015-0871-6 25801513 PMC4715123

[88] Pandharipande PP, Girard TD, Jackson JC, Morandi A, Thompson JL, Pun BT, et al. Long-Term Cognitive Impairment after Critical Illness for the BRAIN-ICU Study Investigators*. N Engl J Med [Internet]. 2013;369:1306–16. Available from: http://www.nejm.org/doi/pdf/10.1056/NEJMoa1301372 24088092 10.1056/NEJMoa1301372 PMC3922401

[89] Francis J, Kapoor WN. Prognosis after Hospital Discharge of Older Medical Patients with Delirium. J Am Geriatr Soc [Internet]. 1992 Jun;40(6):601–6. Available from: http://doi.wiley.com/10.1111/j.1532-5415.1992.tb02111.x 1587979 10.1111/j.1532-5415.1992.tb02111.x

[90] Dziegielewski C, Skead C, Canturk T, Webber C, Fernando SM, Thompson LH, et al. Delirium and Associated Length of Stay and Costs in Critically Ill Patients. Tran QK, editor. Crit Care Res Pract. 2021 Apr;2021:1–8. doi: 10.1155/2021/6612187 33981458 PMC8088381

